# How service delivery implementation strategies can contribute to attaining universal health coverage: lessons from polio eradication using an implementation science approach

**DOI:** 10.1186/s12889-022-13681-0

**Published:** 2022-06-30

**Authors:** Adetoun Olateju, Michael A. Peters, Ikponmwosa Osaghae, Olakunle Alonge

**Affiliations:** 1 Department of International Health, Bloomberg School of Public Health Johns Hopkins University, 615 N Wolfe Street, E8140, Baltimore, MD 21205 USA; 2 Department of Epidemiology, Human Genetics and Environmental Sciences, University of Texas Health Science Center at Houston, Houston, TX USA

**Keywords:** Service delivery strengthening, Universal health coverage, Polio eradication, Implementation contributors, Primary health care, Implementation science

## Abstract

**Background:**

Improving service delivery is a key strategy for achieving service coverage, one of the two components of universal health coverage (UHC). As one of the largest global public health initiatives, individuals involved with the Global Polio Eradication Initiative (GPEI) have learned many important lessons about service delivery. We identified contributors and challenges to delivering health services at national and subnational levels using experiences from the GPEI. We described strategies used to strengthen service delivery and draw lessons that could be applicable to achieving UHC.

**Methods:**

Online cross-sectional surveys based on the Consolidated Framework for Implementation Research (CFIR) domains and socioecological model were conducted from 2018–2019. Data were analyzed using an embedded mixed methods approach. Frequencies of the contributors and challenges to service delivery by levels of involvement were estimated. Chi-square tests of independence were used to assess unadjusted associations among categorical outcome variables. Logistic regressions were used to examine the association between respondent characteristics and contributors to successful implementation or implementation challenges. Horizontal analysis of free text responses by CFIR domain was done to contextualize the quantitative results.

**Results:**

Three thousand nine hundred fifty-five people responded to the online survey which generated 3,659 valid responses. Among these, 887 (24.2%) reported involvement in service delivery at the global, national, or subnational level with more than 90% involved at subnational levels. The main internal contributor of strengthened service delivery was the process of conducting activities (48%); working in frontline role had higher odds of identifying the process of conducting activities as the main internal contributor (AOR: 1.22, *p* = 0.687). The main external contributor was the social environment (42.5%); having 10–14 years of polio program implementation was significantly associated with identifying the social environment as the main external contributor to strengthened service delivery (AOR: 1.61, *p* = 0.038). The most frequent implementation challenge was the external environment (56%); working in Eastern Mediterranean region was almost 4 times more likely to identify the external environment as the major challenge in service delivery strengthening (AOR:3.59, *p* < 0.001).

**Conclusion:**

Priority actions to improve service delivery include: adopt strategies to systematically reach hard-to-reach populations, expand disease-focused programs to support broader primary healthcare priorities, maximize community outreach strategies to reach broader age groups, build community trust in health workers and develop multisectoral leadership for collaboration. Achieving UHC is contingent on strengthened subnational service delivery.

**Supplementary Information:**

The online version contains supplementary material available at 10.1186/s12889-022-13681-0.

## Background

Achieving Universal Health Coverage (UHC) is one of the Sustainable Development Goals (SDG) targets aimed at ensuring healthy lives and well-being for all ages [1]. It emphasizes access to effective and quality health care by all people and communities without financial hardship [2]. The UHC target has two components: first is the effective coverage of essential health services, and second is preventing health-related catastrophic financial expenditure [3]. The essential health service coverage component of UHC highlights the need for effective health service delivery for all people. Strengthening service delivery involves a two-pronged approach on both the supply-side and demand-side of the health system. The supply-side focuses on capacity building of health workers and improving service readiness, availability, and quality at health facilities. The demand-side involves increasing access of individuals and communities to health services, ranging from social mobilization to create demand for services to bringing health services to beneficiaries in their communities.

Progress towards effective service delivery within UHC is monitored using the service coverage index (SCI) which is computed from selected tracer indicators covering four main categories including reproductive, maternal, newborn and child health, infectious diseases control, non-communicable diseases, and service capacity and access [1]. Globally, SCI increased across all regions between 2000 and 2017, however disparities persist in countries and regions towards attainment of UHC [4]. In 2015, Eastern Asia, North American, and European regions had the highest SCI for essential services while sub-Saharan Africa reported the lowest coverage [4]. Although development assistance for disease-specific global health initiatives have more than quadrupled in the last 20 years (from $6.7B to $29.2B between 2000 and 2019), specific funding for health system strengthening which includes UHC barely doubled (from $2.7B to $5.6B) in the same period [5], and the evidence on the contribution of vertical global public health programs, [6] to support countries in reaching UHC goals is ambiguous [7–10].

The Global Polio Eradication Initiative (GPEI) is one of the largest and longest continuous global public health initiative spanning over the last three decades [11]. It was launched in 1988 as a roadmap for the eradication of polio [12]. Since then, there has been substantial reduction in the global incidence of polio with eradication of wide polio virus (WPV) types 2 and 3. Between January and August 2021, only 24 cases of WPV type 1 were reported [13]. Since its inception, investments in the polio eradication initiative (PEI) have contributed to strengthening service delivery [14–16]. These efforts have led to increased routine immunization (RI) coverage in affected regions, and integration of other categories of UHC tracer indicators with polio programs [15, 17, 18]. For example, immunization coverage for measles and BCG improved between 1996 and 2014 in countries in the African region with significant GPEI presence [15]. During this period, Nigeria and Democratic Republic of Congo (DRC) reported over threefold increases in DPT-3 vaccination coverage. Moreover, GPEI efforts have contributed to broader health service delivery efforts through capacity building, micro-planning and disease surveillance [15, 17, 19].

Conversely, the GPEI has been shown to disrupt health systems especially in countries with weaker health system infrastructure by operating in siloes, manifesting as misalignment between GPEI and the health priorities and systems of low-and middle-income countries [8]. This was illustrated as conflicts between local community demands and polio immunization targets, [20] lack of cohesive social mobilization and communication between polio eradication initiative and the expanded program on immunization and diverting operating capacity and human resources from broader health system goals to polio eradication via introduction of unsustainable financial incentives [8, 21]. Service disruption and public dissatisfaction were observed in districts where several polio campaigns were conducted per year as community members perceived an unequal focus on polio over other priority health needs, with equivocal impact of GPEI on coverage of DPT-3 and skilled birth attendance [7]. The seemingly narrow mandate of the program is also a major driver of fragmentation, one of the main challenges in achieving UHC [9].

Despite the various lessons on how the GPEI has interacted with the health system in different settings, and the importance of achieving UHC goals for global health, there has not been a systematic assessment of how programmatic assets from the GPEI can be leveraged for achieving UHC goals, especially access to and coverage of essential health services. Such an assessment will provide a roadmap for integration of polio assets into routine health systems and help inform future global health program implementation to advance the attainment of UHC. The Synthesis and Translation of Research and Innovations from Polio Eradication (STRIPE) research consortium led by Johns Hopkins Bloomberg School of Public Health, consists of eight institutional partners drawn from different geographies that have experienced intense polio eradication investments and activities over the past 10–20 years, and is mapping, contextualizing and documenting the knowledge, resources and lessons learned from polio eradication programs drawing from the perspectives of multilevel implementers (global, national and sub-national) using implementation science research methods [22]. The objective of this paper is to document the implementation strategies used to strengthen service delivery system, and the implementation challenges experienced by health workers across global, national and subnational levels of the GPEI.

## Methods

Data were obtained from a cross-sectional online survey that was conducted to map global and country-level implementation approaches and experiences (tacit knowledge) of the polio eradication program. We used an embedded mixed-methods design [23] where short qualitative responses were collected to enhance the interpretation of the quantitative responses within the same survey questionnaire. The survey drew respondents who identified as working or having previously worked on polio eradication activities for at least 12 consecutive months between 1988 and 2019. The knowledge survey was designed to systematically assess and harmonize the lessons learned in the implementation of polio eradication activities across multiple regions, roles, and years of experience [24]. The online questionnaire was translated and back-translated in nine languages, and was pilot tested with local respondents to ensure that key concepts were understood. Survey participants were from seven low-and-middle countries (Afghanistan, Bangladesh, the Democratic Republic of Congo, Ethiopia, India, Indonesia, Nigeria) and from the global level across member organizations of the GPEI and international non-governmental organizations with experiences in GPEI in additional countries [24]. Details on the countries and World Health Organization (WHO) regions where survey respondents were drawn are described in supplementary table 1 (see additional file 1). Respondents provided information about the internal contributors, external contributors, implementation challenges, facilitators and barriers they encountered during implementation, based on the CFIR framework, supplemented by the socioecological model [22, 25]. The methods for identifying eligible survey respondents and conducting the survey were described in a separate publication [24].

An online questionnaire was administered to participants and data collection was from August 2018 to April 2019. Respondents provided information across the specific objectives of the polio eradication program that they were involved in, namely resource mobilization, partnership development, monitoring & evaluation, strategy development, vaccination, surveillance, communication, and service delivery. These objectives were developed and defined using implementation science frameworks, translated to local languages and refined based on results of pilot testing [24–26]. This paper focuses on respondents who were involved with implementing activities geared towards strengthening service delivery systems – defined as activities that enable vaccination at the right time, for the right populations in the PEI implementation context. Activities included developing infrastructure; recruiting, training, and supervising personnel; strengthening supply chains, and administering vaccines to recipients. Some of these activities were vertically provided and others were embedded within the health services system.

## Measures

### Sociodemographic variables

The years of experience of respondents in 5-year categories were included. Organizational affiliations were classified as government workers, non-profit organizations, member organizations of the Global Polio Eradication Initiative (GPEI) comprising the WHO, UNICEF, Bill & Melinda Gates Foundation, US Centers for Disease Control and Rotary International. The level of organizational involvement whether global, national, or subnational were indicated. Subnational level of involvement was defined as those who implemented polio program activities at state, province, district, or subdistrict levels. The specific roles of respondents such as EPI officer, program manager, surveillance officer, or frontline health worker were also included in addition to the role in which the respondent spent the most time. The WHO region (Eastern Mediterranean, Sub-Saharan Africa, South-East Asia, Western Europe, The Americas, Western Pacific), up to 3 activities performed in respondent’s primary role, and years spent in that role were recorded.

### Outcome variables

The outcomes of interest are internal and external contributors to successful service delivery and implementation challenges affecting service delivery. Both the factors that are within control of intervention design (internal contributors) and those that are influential within an operating environment (external contributors) are important to understand and contextualize when synthesizing lessons learned [25]. Respondents selected the most important internal and external contributors (based on the Consolidated Framework for Implementation Research (CFIR) constructs) to their program’s success in strengthening delivery systems. They also selected the CFIR constructs in which they experienced significant challenges when carrying out health services delivery strengthening activities for GPEI, e.g., providing human resources support and expanding access for ambulatory care, maternal and child health services, and routine immunization services. The definitions of these constructs are provided in Table 1.

**Table 1:** Definitions of contributors and challenges to successful strengthening of service delivery systems using CFIR constructs

Internal contributors	
Individual characteristics	High levels of individual knowledge, self-efficacy, higher likelihood to provide sustained support through stages of change and good perception of the organization
Organizational settings	Clear organizational structure with adequate personnel, strong formal and informal communication networks, healthy organizational culture, presence of leadership readiness for implementation
GPEI program characteristics	Perception of internal vs external source of the interventions, perception of evidence strength and quality, relative advantage of polio program vs other programs, trialability, perceived complexity of implementation, design quality, costs of implementation and opportunity costs
Process of conducting the activities	Stages of the implementation process: detailed planning, engaging with relevant stakeholders, good execution of activities, established mechanisms for monitoring and evaluating program progress and quality
**External contributors**	
Political	Political climate accepting of polio eradication activities, political structure conducive to coordinated action
Economic	Sufficient revenue to fund activities
Social	Social norms around immunization in communities where polio eradication activities were implemented
Technological	Infrastructure or technological advances
**Implementation challenges:** consisted of internal and external contributors that impeded national and subnational implementation of the polio program	
Individual characteristics	Low levels of individual knowledge, lack of self-efficacy, lower likelihood to provide sustained support through stages of change and poor perception of the organization
Organizational settings	Weak organizational structure with inadequate personnel, limited formal and informal communication networks, poor organizational culture, absence of leadership readiness for implementation
GPEI program characteristics	Perception of internal vs external source of the interventions, perception of evidence strength and quality, relative advantage of polio program vs other programs, trialability, perceived complexity of implementation, design quality, costs of implementation and opportunity costs
Process of conducting the activities	Stages of the implementation process: lack of advanced planning, challenges of attracting and engaging with relevant stakeholders, poor execution of activities, difficulty with monitoring and evaluating program progress and quality
External settings	Challenges related to the external political, economic, social, and technological environments

Source: Alonge et al. Synthesis and translation of research and innovational from polio eradication (STRIPE): initial findings from a global mixed methods study. BMC Public Health 20, 1176 (2020). https://doi.org/10.1186/s12889-020-09156-9

Respondents further described the major challenges they experienced in strengthening delivery systems, solutions used to overcome these challenges, and any unintended consequences in open text responses. For these implementation challenges and based on the category identified (Table 1), further questions were asked to identify the root cause of each challenge. For example, if individual characteristics were selected as a challenge to strengthening health services delivery, respondents would be asked if the individual characteristics were related to personal attributes such as individual level of knowledge, self-efficacy, or stage of change. If the process of program implementation was selected as an implementation challenge, further questions around implementation processes such as planning, engaging, executing, or evaluating were asked.

### Data analysis

Descriptive characteristics of respondents were analyzed by level of involvement (i.e., at the global, national, sub-national levels) in service delivery implementation. Frequencies of the contributors and challenges to implementing service delivery by levels of involvement were estimated. The most frequent factors identified as contributors and challenges were treated as categorical outcome variables. Chi-square tests of independence were used to assess unadjusted associations among the categorical outcome variables. Bivariate (see additional file 1) and multivariable logistic regression models were used to estimate the association between the sociodemographic characteristics and various contributors of service delivery systems, and to assess the association between implementation challenges and sociodemographic variables. Adjusted odds ratios were used to determine the magnitude and significance of the associations. Quantitative data analysis was conducted using Stata version 14. (StataCorp. 2015. Stata Statistical Software: Release 14. College Station, TX: StataCorp LP.)

Free text responses within the survey were analyzed to enhance the interpretation of the quantitative results by providing illustrative examples. All responses were translated to English by the study team. The median response contained 96 characters and responses ranged between 0 and 900 characters. Horizontal analysis was conducted on free text responses by CFIR domain to elucidate themes within each of the contributors and challenges to strengthening service delivery across respondents.

### Ethical approval

The online survey was approved by Johns Hopkins School of Public Health’s Institutional Review Board. All respondents gave informed written consent before starting the online questionnaire.

## Results

Three thousand nine hundred fifty-five people responded to the online survey and 3,659 valid responses were analyzed. Among these, 887 (24.2%) implemented activities to strengthen service delivery systems. Table 2 describes respondent characteristics by level of implementation involvement for these 887 individuals. The participating countries and regions of valid survey responses are detailed in supplementary table 1 (Additional file 1). Three percent reported involvement with implementing activities at the global level, 7% reported involvement at the national level, and 90% of respondents implemented polio program activities at the subnational level. Survey respondents implementing service delivery at the subnational level were more frequently working for government (52.5%) and given their role within the government health services delivery system, we categorize their health services delivery strengthening activities as embedded within the health services delivery system. Survey respondents at the global level were more frequently working at a GPEI partner organization (46.2%).

**Table 2:** Respondent characteristics by level of implementation involvement (*n* = 887)

	% within levels of involvement (95% CI)		
	Global	National	Subnational
Respondent characteristics	(*n* = 26)	(*n* = 63)	(*n* = 798)
Organizational affiliation			
GPEI partners	46.2 (28.0–65.3)	27.0 (17.4—39.3)	15.6 (13.2–18.3)
Government	27.0 (13.2–47.2)	30.2 (20.1–42.6)	52.5 (49.0–56.0)
Implementers	7.7 (1.9–26.7)	20.6 (12.3–32.5)	18.6 (16.0–21.4)
Research and others	19.2 (8.1–39.2)	22.2 (13.6–34.2)	13.3 (11.1–15.9)
Years of experience			
0–4	69.2 (49.0—84.0)	34.9 (24.1—47.5)	24.1 (21.2–27.2)
5–9	11.5 (3.7–30.8)	19.1 (11.1–30.7)	30.3 (27.2–33.6)
10–14	7.7 (1.9–26.7)	22.2 (13.6–34.2)	20.2 (17.5–23.1)
15–19	7.7 (1.9–26.7)	17.5 (9.9–29.0)	15.3 (13.0–18.0)
20 and above	3.9 (0.5–23.5)	6.3 (0.2–15.9)	10.2 (8.3–12.5)
Role			
Advisory	7.7 (1.9–26.7)	4.8 (1.5–13.9)	2.5 (1.6–3.9)
Management	19.2 (8.1–39.2)	6.3 (2.4–15.9)	3.5 (2.4–5.0)
Supervisory	19.2 (8.1–39.2)	42.9 (31.2–55.4)	34.1 (31.0–37.5)
Frontline	23.1(10.6–43.2)	20.7 (12.1–32.5)	35.3 (32.1–38.7)
Other	31 (16.0–51.0)	25.4 (16.1–37.6)	24.6 (21.7–27.7)
Region			
Africa	63.6 (41.8–81.0)	58.9 (45.6–71.1)	57.6 (53.9–61.3)
South-East Asia	0	12.5 (6.0–24.1)	25.5 (22.4–28.9)
Eastern Mediterranean	31.8 (15.7–53.9)	25.0 (15.3–38.1)	12.6 (10.3–15.3)
Western hemisphere(Europe, America, Western Pacific regions)	4.5 (0.6–27.1)	3.6 (0.9–13.4)	4.2 (2.9–6.0)

*95% CI* 95% confidence interval

GPEI partners: WHO, UNICEF, Bill & Melinda Gates Foundation, US Centers for Disease Control, Rotary International

Frontline roles: polio vaccinators, surveillance officers; Subnational level of involvement: state, province, district or subdistrict. Western hemisphere: Europe, Americas and Western Pacific regions

Fifty-six percent of respondents had less than 10 years of experience implementing polio programs. About 70% of the subnational level respondents were in frontline and supervisory roles, which were those who could respond directly to service delivery. Most respondents worked in the Africa region (58%), 24% worked in Southeast Asia, 14% worked in Eastern Mediterranean and only 4% worked in the western hemisphere.

Survey respondents identified the biggest internal contributor to strengthening health systems as the process of conducting activities (48%). Indicative examples were drawn from free-text responses made by respondents (Table 3). The biggest external contributor was the social environment (42.5%) while external settings (55.8%) were identified as the biggest challenge followed by challenges related to program implementation (22.5%).

**Table 3:** Internal and external contributors and implementation challenges of strengthening service delivery across respondents

Biggest internal contributor to health services delivery strengthening activities (*n* = 848)	Freq. (%)	Illustrative Example
Process of conducting activities	408 (48.11%)	Specific strategies to systematically reach difficult populations, including microplanning
Individual characteristics	157 (18.51%)	Motivated and knowledgeable health workers and volunteers
Organizational settings	148 (17.45%)	Collaboration and effective coordination among NGOs, government, and donors
Polio program characteristics	135 (15.92%)	Relative ease of storing and administering OPV
Biggest external contributor to health services delivery strengthening activities (*n* = 843)		
Social environment	358 (42.47%)	High levels of trust and community awareness towards health workers and vaccines
Political environment	221 (26.22%)	Strong political leadership and support, overall peace and stability in the country
Economic environment	167 (19.81%)	Sufficient and timely funding
Technological environment	79 (9.37%)	Expansion of mobile phone networks enhanced coordination and delivery
Other environment	18 (2.14%)	Accessible geographies
Challenges to strengthening health service delivery (*n* = 830)		
External settings	463 (55.78%)	Security challenges, community resistance
Implementation	187 (22.53%)	Lack of adaptation to meet local needs and context
Individual	82 (9.88%)	Lack of confidence or motivation among health workers
Organizational	62 (7.47%)	Competing priorities, and lack of accountability mechanisms
Polio program-related	36 (4.34%)	National immunization days and supplementary immunization activities do not support routine immunization

Across all levels of implementation, over 40% of respondents identified the process of conducting activities as the greatest internal contributor of success. (Table 4) At the sub-national level, the second biggest internal contributor was individual characteristics. Externally, the social environment was the most important external contributor of success across all respondents (approximately 40%) at all levels of involvement. Interestingly, at the global level, both political and economic environments (25%) were the second important external contributors of success. There was a switch at national and sub-national levels, where at national level, similar to the global level, the economic environment was the second highest contributor (25%) while at the sub-national level, the political environment was the second highest contributor (26.3%), however these differences were not statistically significant. External environment challenges were overwhelmingly the highest contributor of implementation challenges ranging from 49% at national level to 73% at global level. At national and sub-national levels, 25% and 23% of respondents respectively noted that challenges linked to the process of implementation was the second highest implementation challenge.

**Table 4:** Contributors and challenges by level of implementation involvement

	% within levels of involvement (95% CI)		
Contributors and challenges	Global	National	Subnational
	(*n* = 24)	(*n* = 56)	(*n* = 767)
Internal contributors (chi-square: 9.22; p:0.162)			
Individual characteristics	16.7 (6.3–37.5)	21.4 (12.5–34.2)	18.3 (15.7–21.2)
Organizational settings	4.17 (0.6–25.2)	23.2 (13.9–36.1)	17.5 (14.9–20.3)
Polio program characteristics	33.3 (17.3–54.4)	12.5 (6.0–24.1)	15.7 (13.2–18.4)
Process of conducting activities	45.8 (27.1–65.8)	42.9 (30.5–56.1)	48.6 (45.1–52.2)
External contributors (chi-square: 5.82; p:0.667)			
Political environment	25 (11.5–46.2)	19.6 (11.2–32.2)	26.8 (23.7–30.0)
Economic environment	25 (11.5–46.2)	25 (15.3–38.1)	19.3 (16.6–22.3)
Social environment	41.7(23.7–62.1)	39.3 (27.3–52.7)	42.6 (39.2–46.2)
Technological environment	8.3 (2.0–28.6)	10.7 (4.9–22.0)	9.3 (7.4–11.6)
Implementation challenges (chi-square: 8.60; *p* = 0.377)			
Individual challenges	18.2 (6.8–40.3)	13.2 (6.4–25.4)	9.4 (7.5–11.7)
Organizational challenges	0	7.6 (2.8–18.6)	7.7 (6.0–9.8)
GPEI-related challenges	0	5.7 (1.8–16.3)	4.4 (3.1–6.1)
Implementation challenges	9.1(2.2–30.7)	24.5 (14.7–38.0)	22.8 (19.9–26.0)
External challenges	72.7 (50.5–87.5)	49.1 (35.8–62.4)	55.7 (52.1–59.2)

### Multivariable logistic regression

Tables 5 and 6 explored the association between the internal (Table 5) and external (Table 6) contributors of success to health services delivery strengthening activities within the polio program and sociodemographic variables. Table 7 assessed the association between implementation challenges to health services delivery strengthening activities within the polio program and characteristics of respondents**.**

**Table 5:** Factors associated with internal contributors to strengthening service delivery at national and subnational levels

Main internal contributor: Process of implementation	Odds Ratio	*p*-value	[95% Conf. Interval]
Organizational affiliation			
GPEI partner institutions (ref)	1		
Government	1.127	0.596	[0.724, 1.754]
Implementers	1.156	0.581	[0.690, 1.939]
Others (researchers, etc.)	1.089	0.772	[0.613, 1.932]
Years of experience			
0—4 (ref)	1		
5—9	0.697*	0.079	[0.466, 1.042]
10—14	0.533***	0.007	[0.337, 0.841]
15—19	0.647*	0.083	[0.396, 1.058]
20 +	0.680	0.181	[0.387, 1.197]
Role			
Advisory (ref)	1		
Management	0.722	0.598	[0.215, 2.426]
Supervisory	0.642	0.359	[0.249, 1.656]
Frontline	1.221	0.683	[0.469, 3.174]
Other	1.027	0.957	[0.392, 2.69]
Region			
Africa (ref)	1		
South-East Asia	1.157	0.434	[0.803, 1.667]
Eastern Mediterranean	0.569**	0.023	[0.35, 0.925]
Western Hemisphere	1.328	0.457	[0.628, 2.808]
Number of observations	743		

^***^ *p* < .01, ** *p* < .05, * *p* < .1

**Table 6:** Factors associated with external contributors to strengthening service delivery at national and subnational levels

Main external contributor: Social environment	Odds Ratio	*p*-value	[95% Conf. Interval]
Organizational affiliation			
GPEI partner institutions (ref)	1		
Government	1.102	0.665	[0.710, 1.712]
Implementers	1.099	0.717	[0.659, 1.834]
Others (researchers, etc.)	0.723	0.277	[0.403, 1.298]
Years of experience			
0—4 (ref)	1		
5—9	1.076	0.725	[0.716, 1.617]
10—14	1.611**	0.038	[1.027, 2.528]
15—19	1.305	0.289	[0.798, 2.135]
20 +	0.91	0.725	[0.504, 1.612]
Role			
Advisory (ref)	1		
Management	0.517	0.288	[0.153, 1.745]
Supervisory	0.600	0.288	[0.234, 1.539]
Frontline	0.674	0.416	[0.261, 1.744]
Other	0.648	0.375	[0.249, 1.689]
Region			
Africa (ref)	1		
South-East Asia	0.911	0.621	[0.628, 1.321]
Eastern Mediterranean	1.610**	0.043	[1.015, 2.554]
Western Hemisphere	0.776	0.521	[0.358, 1.683]
Number of observations	743		

^***^ *p* < .01, ** *p* < .05, * *p* < .1

**Table 7:** Factors associated with challenges of strengthening service delivery at national and subnational levels

Main delivery challenge: External environment	Odds Ratio	*p*-value	[95% Conf. Interval]
Organizational affiliation			
GPEI partner institutions (ref)	1		
Government	1.08	0.751	[0.673, 1.731]
Implementers	0.737	0.281	[0.423, 1.284]
Others (researchers, etc.)	1.222	0.524	[0.659, 2.265]
Years of experience			
0—4 (ref)	1		
5—9	0.657*	0.054	[0.428, 1.008]
10—14	0.601**	0.039	[0.371, 0.974]
15—19	0.574**	0.036	[0.341, 0.964]
20 +	0.673	0.19	[0.372, 1.217]
Role			
Advisory (ref)	1		
Management	8.095***	0.003	[2.049, 31.974]
Supervisory	4.082***	0.008	[1.437, 11.596]
Frontline	4.260***	0.007	[1.487, 12.199]
Other	3.938**	0.012	[1.359, 11.405]
Region			
Africa (ref)	1		
South-East Asia	0.66**	0.034	[0.449, 0.969]
Eastern Mediterranean	3.588***	< 0.001	[2.043, 6.302]
Western Hemisphere	1.386	0.425	[0.622, 3.087]
Number of observations	700		

^***^ *p* < .01, ** *p* < .05, * *p* < .1

Holding other variables constant, those who worked for 5 or more years on polio implementation had significantly lower odds of identifying the process of polio implementation as the main internal contributor, compared to those who worked for 0–4 years. Regionally, those who implemented polio programs in the Eastern Mediterranean region had significantly lower odds of identifying the process of polio implementation as the main internal contributor of success, while those who worked in Southeast Asia and the Western Hemisphere had higher odds of identifying the implementation process as the main internal contributor of success, compared to those implementing in the Africa region.

Those who worked for 5 or more years on polio implementation had higher odds of identifying the social environment as the main external contributor, compared to those who worked for 0–4 years, except those who worked for 20 or more years. Regionally, those who implemented polio programs in the Eastern Mediterranean region had significantly higher odds of identifying the social environment as the main external contributor of success, compared to their counterparts implementing in the Africa region, after adjusting for all other variables.

Regarding the factors associated with the challenges of strengthening service delivery, compared to those with 0–4 years of experience, those with 5 or more years of experience had significantly lower odds of selecting the external environment as the main challenge, except those who worked for 20 or more years. All those who indicated working in national and sub-national management, supervisory and frontline roles had at least four times significantly higher odds of identifying the external social environment as the biggest challenge to service delivery, compared to those in advisory roles. Regionally, those who implemented polio programs in the Eastern Mediterranean region had almost four times significantly higher odds, while those working in South-East Asia had significantly lower odds of identifying the external social environment as the main implementation challenge, compared to their counterparts implementing in the Africa region, after adjusting for all other variables.

## Discussion

### Major internal contributor to service delivery

Strengthening service delivery on the supply side of the health system involves improving quality and availability of services provided at point-of-care in health facilities, training, skill building and task shifting. On the demand side, it involves removing barriers to access to health services such as bringing health services to the most vulnerable populations. Our study showed that the process of implementing activities was the most important internal contributor to strengthening service delivery. We found strategies aimed at systematically increasing access to healthcare services to reach remote and vulnerable populations included microplanning, community inclusion and use of geographic information systems. Some of the strategies previously documented in other studies included the use of innovative technology like geographic information systems to identify chronically unvaccinated children, [15] inclusion of nomadic communities to develop immunization plans, [15] transit vaccinations at major transportation hubs and markets, [27] and using military personnel as vaccinators during supplementary immunization activities in regions of violent conflicts [28]. These strategies subsequently improved not only polio immunization coverage, but also had spillover effects that improved service delivery and contributed to increased routine immunization coverage in regions where they were implemented [15].

In our study, some survey respondents cited the training of frontline and community health workers who implemented the social mobilization network (SMNet) program was not limited to polio immunization activities in India. Other studies demonstrated SMNet health workers were also trained to promote general maternal and child health including tracking children’s complete vaccination history, home management of childhood diarrhea, household hygiene, and breastfeeding promotion [17, 29]. Such programs that extended beyond polio immunization to address broader public health priorities contributed to strengthening service delivery. Some studies showed that SMNet program continued to support routine childhood immunization, and improved primary health care service delivery in Uttar Pradesh, Bihar and West Bengal [14, 17, 29]. In Indonesia, the polio program was integrated in the existing sub-national government institutions for immunization [30]. At the community level, integrated health services posts (“Posyandu*”*) delivered polio immunization along with maternal and child health and nutrition, which may have contributed to stronger health service delivery [30]. We found significantly lower odds of identifying polio implementation process as a main internal contributor for strengthening service delivery in Eastern Mediterranean region. For example, in Pakistan, polio supplementary immunization activites were criticized as being disjointed from routine childhood immunization services and other primary health care services [31]. Similarly in Afghanistan, Rodriguez et al. found that the extensive separation of polio program from routine immunization significantly impacted service delivery strengthening at the sub-national levels [8].

There is documented evidence on the impact of PEI in improving RI, and the integration of Vitamin A supplementation and RI with polio activities [11]. In addition, the GPEI’s infrastructural assets, investments and impact could be applicable to other aspects of population health. The 19^th^ polio Independent Monitoring Board (IMB) report showed that countries in Africa and East Mediterranean used their existing polio eradication infrastructure ranging from human resources to data management, communication and surveillance worth over 100 M USD, as part of their COVID-19 pandemic response [32]. Similarly, during the 2014–2016 West Africa Ebola outbreak, Nigeria deployed existing polio assets for prompt detection and quickly quelled the spread [33, 34].

Beyond the intermittent emergency response applications, polio presents a unique opportunity to re-design service delivery using an integrated, people-centered approach. Importantly, this approach takes advantage of the existing knowledge and investments from the PEI. For example, similar GIS techniques used for micro-planning during polio eradication activities [35, 36] can also be utilized for planning non-communicable disease screening and targeted maternal health interventions. Additional interventions can also be integrated into polio activities. For example, when parents and caregivers bring children for vaccination during supplementary immunization days, or during door-to-door campaigns, blood pressure readings and finger prick tests to screen for hypertension and diabetes, respectively, could be integrated into these visits. Countries can leverage GPEI infrastructural assets and knowledge to bring these services to the doorsteps of vulnerable populations for prioritized interventions.

### Main external contributor and implementation challenge

We found the most important external contributor to strengthen service delivery was the social environment, especially among those whose roles interfaced directly with communities and beneficiaries. Our study found the elements of the social environment included developing high levels of trust in health workers, transparency in the vaccination process and building community awareness. Conversely, the external environment was identified as the main implementation challenge hampering the strengthening of service delivery. We found respondents in Eastern Mediterranean region had significantly higher odds of identifying the external environment as the most important implementation challenge compared to the reference region. In the two remaining polio-endemic countries, the complex external environment challenges were similarly characterized. Respondents cited security challenges, and community resistance to polio eradication interventions as the main forms of implementation challenges experienced. A recent review found insecurity and conflicts remain a persistent barrier to service delivery in Pakistan, [31] and Afghanistan continues to grapple with complex challenges of prolonged religious and ethnic conflicts, mistrust in health workers and political upheavals [37]. Community resistance is often the manifestation of a lack of community trust, which was a major challenge in the eradication of polio according to the polio IMB [32]. In high-conflict and high-risk states like Pakistan, lack of trust in polio vaccinators was also identified as a major challenge impeding polio eradication [38].

Strategies to mitigate community resistance while circumventing security challenges included shorter immunization days (‘hit-and-run’) and using elderly traditional birth attendants as part of permanent community health teams in high-conflict areas in Nigeria [39]. In Ethiopia, the use of community volunteers (CVs) to secure community trust by addressing misconceptions about polio vaccines while helping implementers identify routes to hard-to-reach, border communities was effective [40]. In India, the social mobilization networks created communication channels to reach high-burden communities that were typically impoverished, laden with other non-polio priorities, had cultural/religious conservatism, and generally distrustful of government [41].

Building trust between health workers and communities requires a medium to long-term approach before achieving success [40]. It entails contextualizing social mobilization and community awareness activities in individual communities to improve buy-in and participation. Unlike smallpox, or COVID-19 that involved one or two vaccination shots, polio eradication requires multiple contacts with the health system before vaccination, similar to other public health services like RI, hypertension or diabetes management, cancer screening, antenatal care, and other services that make up the essential health services package of universal health coverage in many countries. Thus, when communities need multiple, repeated contacts with the health system, building trust requires constant communication with consistent messaging, while modeling health promoting behaviors, and understanding cultural norms. Health care providers and community health workers who are often the first contacts in the health system need to transform beyond providing health services (competence and knowledge) to become trust agents (morality and compassion for the people they serve) fostering community ownership and engagement while serving as pillars to strengthen service delivery [41]. This is fundamental to one of the primary health care principles to ensure communities can afford to sustain health at all levels of their development [42].

### Implications for universal health coverage

Reviewing the health system’s capacity to deliver on UHC priorities should include information on service delivery [43] because making progress towards national UHC goals is contingent on strengthening service delivery [3]. Global programs like GPEI can both contribute and hamper UHC efforts at global, national, and subnational levels. At subnational levels, they hamper UHC efforts when programs are not aligned with the community’s priorities and contribute to UHC when the programs are a direct response to the community’s needs and priorities. In some communities, these programs are the healthcare lifeline when health budgets are insufficient. The assets from polio eradication programs including resources and dedicated manpower at the subnational levels can be leveraged to support broader systemic UHC efforts like improving availability and quality of service delivery which has direct impact on the population.

The need for a comprehensive primary health care delivery model that promotes interaction by health care professionals and the community in the formulation and execution of health goals at personal and community level have been previously documented [44]. This model which is based on community identified needs is framed on program flexibility, adaptation, interdisciplinary partnership, on-going evaluation, and adjustment of services to meet emerging needs [44]. The integration of GPEI with other primary care services have been shown to expand coverage for maternal, newborn and child health services in hard-to-reach communities which are unlikely to have contact with basic health care [18]. In Nigeria, this model led to increased awareness on other vaccine preventable diseases (such as measles, cerebrospinal meningitis, yellow fever, pertussis) and increased access to health services at the nearest health facility [18]. Similarly, following an integrated campaign for insecticide treated nets (ITN) and polio vaccination in Niger, there was substantial increase in ITN ownership with accompanied decrease in inequities between highest and lowest wealth quintiles [45].

Currently, there is a focus on integration of implementation of PEI and RI as evidenced by the recent strategic plans of Gavi Phase V (2021–2025), [46] the upcoming Polio Eradication Strategy (2022–2026), [47] and the launch of the interim Program of Work for Integrated Actions that synergizes GPEI and the Essential Programme on Immunization (EPI) in the context of COVID-19 [48]. Integration needs to extend beyond specific disease programs to diagonal, multi-dimensional, equity-focused integration that incorporates primary health care, effective preventive interventions, and clinical management to strengthen service delivery in the health systems [49]. Afghanistan and Pakistan, the remaining two polio-endemic countries are exemplary as they have documented plans to integrate PEI with other basic primary health care services as a UHC package of essential services [32]. To achieve UHC, intense efforts and resources dedicated to control or eradication of individual disease is insufficient; countries should be supported to build sustainable systems to prevent, detect and effectively respond to disease outbreaks and emerging threats to public health.

In developing and implementing national UHC plans, the external environment should not be overlooked. We found that the external environment was the most significant challenge to strengthening service delivery. This included factors beyond the scope of the health sector alone such as perennial violent conflicts, political upheavals, monumental changes e.g., moving from centralized to decentralized system of governance; all of which would have varying impacts on the demand and supply sides of the health system. Multisectoral expertise beyond the health sector (education, family affairs, youth empowerment, military etc.) should be drawn upon to address the external environment challenges that would otherwise impede implementation and progress towards achieving UHC. In the context of UHC, service delivery needs to be re-imagined for implementing integrated, essential primary healthcare services that requires regular touchpoints and community ownership. To achieve UHC, this integration needs to be expanded beyond pandemic and emergency responses to incorporate basic essential services, leverage synergies provided by global polio programs to maximize capabilities, fill gaps, and transform the biggest implementation challenges into pillars for strengthening service delivery.

### Strengths & limitations

Our study focused on the national and subnational levels of implementation where service delivery happens. Thus, we captured program implementation knowledge that are closest to the communities. There were some limitations in our study. We did not capture the experiences of polio beneficiaries across contexts. Also, the online survey format might have missed some of the ground-level workers in more remote communities, and possible respondents’ bias could not be completely eliminated despite inbuilt checks to minimize contradictory responses. However, our findings were robust to capture experiences across different countries and contexts, and we cross-pollinated responses to identify the most common and largest contributors and challenges in the various polio eradication implementation contexts.

## Conclusion

We suggest the following priority actions to inform policymakers to accelerate progress towards attainment of universal health coverage:

1. Adopt strategies to systematically reach difficult-to-reach, vulnerable populations such as microplanning and leverage innovative technologies like geographic information systems to identify and reach chronically underserved communities or to target pockets of disease outbreaks and chronic illnesses
2. Expand disease specific programs to support broader primary healthcare priorities e.g., routine immunization, surveillance of all diseases with public health significance
3. Foster holistic health integration such that other members of the community can receive some basic healthcare during immunization campaigns e.g., providing blood pressure measurements to parents during supplementary immunization days
4. Invest in building trust between frontline health workers and the communities they serve by ensuring the community has a voice in setting their health priorities

Multisectoral expertise and leadership to address challenges external to the health system with impact on service delivery should be instituted e.g., developing collaborations with other relevant sectors of the economy such as education, youth empowerment.

## Supplementary Information

**Additional file 1.**

## Data Availability

The datasets supporting the findings of this article are available via the Open Science Framework repository: https://osf.io/9ktgu/?view_only=dcaefa4871f94a9cb37ed72c1d29dc4a

## Abbreviations

CFIR: Consolidated Framework for Implementation Research
EPI: Expanded Program on Immunization
GPEI: Global Polio Eradication Initiative
RI: Routine Immunization
SCI: Service Coverage Index
SDG: Sustainable Development Goals
UHC: Universal Health Coverage
WPV: Wild Polio Virus
