# Supplementary material for: How service delivery implementation strategies can contribute to attaining universal health coverage: lessons from polio eradication using an implementation science approach
Source: BMC Public Health. 2022 Jun 30;22:1271. doi: 10.1186/s12889-022-13681-0 (PMC9244363; doi:10.1186/s12889-022-13681-0)
Supplement: Supplementary file 1 — Additional file 1. [file 12889_2022_13681_MOESM1_ESM.docx]

**Additional file 1:**

**Detailed survey respondents’ characteristics**

**Supplementary table 1: Geographic, implementation level and organizational distribution of survey respondents**

| **Country distribution** | Survey respondents with valid responses (N=3,659) | |
| --- | --- | --- |
| Respondents from STRIPE partner countries | Frequency | Percent |
| Afghanistan | 513 | 14.0% |
| Bangladesh | 106 | 2.9% |
| Democratic Republic of Congo | 499 | 13.6% |
| Ethiopia | 101 | 2.8% |
| India | 401 | 11.0% |
| Indonesia | 322 | 8.8% |
| Nigeria | 921 | 25.2% |
| Respondents from global level by experience in countries within WHO regions |  |  |
| AFRO | 358 | 9.8% |
| AMRO | 33 | 0.9% |
| EMRO | 169 | 4.6% |
| EURO | 45 | 1.2% |
| SEARO | 124 | 3.4% |
| WPRO | 67 | 1.8% |

**Bivariate logistic regressions examining the association between sociodemographic variables and outcome measures**

**Supplementary table 2. Internal contributors**

| Main internal contributor: Process of implementation | Odds Ratio | p-value | (95% Conf. Int.) |
| --- | --- | --- | --- |
| Organizational affiliation |  |  |  |
| GPEI partner institutions (ref) | 1 | - |  |
| Government | 1.191 | 0.37 | (0.813,1.746) |
| Implementers | 1.173 | 0.492 | (0.744,1.848) |
| Others (researchers, etc.) | 0.969 | 0.901 | (0.593,1.585) |
| Years of experience |  |  |  |
| 0 - 4 (ref) | 1 | - |  |
| 5 - 9 | 0.696^*^ | 0.052 | (0.483,1.003) |
| 10 - 14 | 0.732 | 0.128 | (0.49,1.094) |
| 15 - 19 | 0.683^*^ | 0.086 | (0.442,1.056) |
| 20+ | 0.81 | 0.411 | (0.489,1.339) |
| Role |  |  |  |
| Advisory (ref) | 1 | - |  |
| Management | 0.746 | 0.596 | (0.253,2.199) |
| Supervisory | 0.701 | 0.414 | (0.3,1.642) |
| Frontline | 1.241 | 0.618 | (0.531,2.902) |
| Other | 0.956 | 0.918 | (0.404,2.262) |
| Region |  |  |  |
| Africa (ref) | 1 | - |  |
| South-East Asia | 1.152 | 0.424 | (0.814,1.629) |
| Eastern Mediterranean | 0.617^**^ | 0.037 | (0.392,0.972) |
| Western Hemisphere | 1.477 | 0.296 | (0.71,3.073) |
| *** p<.01, ** p<.05, * p<.1 | | | |

**Supplementary table 3. External contributors**

| Main external contributor: Social environment | Odds Ratio | p-value | (95% Conf. Int.) |
| --- | --- | --- | --- |
| Organizational affiliation |  |  |  |
| GPEI partner institutions (ref) | 1 | - |  |
| Government | 1.118 | 0.575 | (0.757,1.65) |
| Implementers | 1.271 | 0.307 | (0.802,2.014) |
| Others (researchers, etc.) | 0.836 | 0.49 | (0.503,1.389) |
| Years of experience |  |  |  |
| 0 - 4 (ref) | 1 | - |  |
| 5 - 9 | 1.189 | 0.364 | (0.818,1.73) |
| 10 - 14 | 1.537^**^ | 0.038 | (1.023,2.31) |
| 15 - 19 | 1.229 | 0.361 | (0.789,1.915) |
| 20+ | 0.903 | 0.705 | (0.533,1.531) |
| Role |  |  |  |
| Advisory (ref) | 1 | - |  |
| Management | 0.496 | 0.215 | (0.164,1.502) |
| Supervisory | 0.731 | 0.471 | (0.313,1.711) |
| Frontline | 0.759 | 0.524 | (0.324,1.776) |
| Other | 0.73 | 0.475 | (0.308,1.731) |
| Region |  |  |  |
| Africa (ref) | 1 | - |  |
| South-East Asia | 0.862 | 0.413 | (0.603,1.23) |
| Eastern Mediterranean | 1.674^**^ | 0.02 | (1.083,2.588) |
| Western Hemisphere | 0.801 | 0.568 | (0.375,1.714) |
| *** p<.01, ** p<.05, * p<.1 | | | |

**Supplementary table 4. Implementation challenges**

| Main delivery challenge: External environment | Odds Ratio | p-value | (95% Conf. Int.) |
| --- | --- | --- | --- |
| Organizational affiliation |  |  |  |
| GPEI partner institutions (ref) | 1 | - |  |
| Government | 0.959 | 0.838 | (0.643,1.432) |
| Implementers | 1.042 | 0.864 | (0.648,1.678) |
| Others (researchers, etc.) | 1 | 1 | (0.596,1.677) |
| Years of experience |  |  |  |
| 0 - 4 (ref) | 1 | - |  |
| 5 - 9 | 0.782 | 0.207 | (0.534,1.146) |
| 10 - 14 | 0.728 | 0.139 | (0.479,1.109) |
| 15 - 19 | 0.668^*^ | 0.082 | (0.424,1.053) |
| 20+ | 0.669 | 0.132 | (0.396,1.129) |
| Role |  |  |  |
| Advisory (ref) | 1 | - |  |
| Management | 3.187^*^ | 0.05 | (0.999,10.171) |
| Supervisory | 2.274^*^ | 0.07 | (0.934,5.535) |
| Frontline | 2.314^*^ | 0.064 | (0.951,5.631) |
| Other | 2.523^**^ | 0.045 | (1.021,6.237) |
| Region |  |  |  |
| Africa (ref) | 1 | - |  |
| South-East Asia | 0.693^**^ | 0.049 | (0.481,0.998) |
| Eastern Mediterranean | 3.127^***^ | <0.001 | (1.84,5.314) |
| Western Hemisphere | 1.306 | 0.504 | (0.597,2.856) |
| *** p<.01, ** p<.05, * p<.1 | | | |
